# The miR‐6779/XIAP axis alleviates IL‐1β‐induced chondrocyte senescence and extracellular matrix loss in osteoarthritis

**DOI:** 10.1002/ame2.12529

**Published:** 2025-02-04

**Authors:** Zongchao Li, Aonan Dai, Xiaoxiang Fang, Kexing Tang, Kun Chen, Peng Gao, Jingyue Su, Xin Chen, Shengwu Yang, Zhenhan Deng, Liangjun Li

**Affiliations:** ^1^ Department of Orthopaedics, The Affiliated Changsha Central Hospital, Hengyang Medical School University of South China Changsha Hunan China; ^2^ Department of Orthopaedic Surgery The First Affiliated Hospital of Wenzhou Medical University Wenzhou Zhejiang China; ^3^ Geriatrics Center The First Affiliated Hospital of Wenzhou Medical University Wenzhou Zhejiang China

**Keywords:** chondrocyte, miR‐6779, osteoarthritis, senescence, X‐linked inhibitor of apoptosis protein

## Abstract

**Background:**

Osteoarthritis (OA) is a long‐term degenerative joint disease worsening over time. Aging and chondrocyte senescence contribute to OA progression. MicroRNAs have been confirmed to regulate different cellular processes. They contribute to OA pathology and may help to identify novel biomarkers and therapies for OA.

**Methods:**

This study used bioinformatics and experimental investigations to analyze and validate differentially expressed miRNAs in OA that might affect chondrocyte apoptosis and senescence.

**Results:**

miR‐6779 was found to be significantly down‐regulated in OA. Seventy‐six of the predicted and miR‐6779 targeted genes and the OA‐associated disease genes overlapped, and these were enriched in cell proliferation, cell apoptosis, and cell cycle. miR‐6779 overexpression remarkably attenuated IL‐1β effects on chondrocytes by reducing MMP3 and MMP13 levels, promoting cell apoptosis, suppressing cell senescence, and increasing caspase‐3, caspase‐9 and reducing P16 and P21 levels. miR‐6779 targeted inhibition of X‐linked inhibitor of apoptosis protein (XIAP) expression. XIAP knockdown partially improved IL‐1β‐induced chondrocyte senescence and dysfunction. Lastly, when co‐transfected with a miR‐6779 agomir, the XIAP overexpression vector partially attenuated the effects of miR‐6779 overexpression on chondrocytes; miR‐6779 improved IL‐1β‐induced senescence and dysfunction in chondrocytes through targeting XIAP.

**Conclusion:**

miR‐6779 is down‐regulated, and XIAP is up‐regulated in OA cartilage and IL‐1β‐treated chondrocytes. miR‐6779 inhibits XIAP expression, thereby promoting senescent chondrocyte cell apoptosis and reducing chondrocyte senescence and ECM loss through XIAP.

## Graphical abstract

Bioinformatics analysis of the differentially expressed miRNA miR‐6779 in GSE105027 and GSE175961 samples predicted a potential downstream target gene XIAP. Human primary chondrocytes were collected and incubated with interleukin‐1 beta (IL‐1β) to simulate osteoarthritis (OA) in an in vitro environment to establish an OA chondrocyte model. By experimentation, it was found that miR‐6779 was expressed at a higher level in IL‐1β‐induced OA chondrocytes, and that miR‐6779 could target and inhibit XIAP, thereby promoting apoptosis in IL‐1β‐induced OA chondrocytes, inhibiting senescence and the degradation of extracellular matrix.

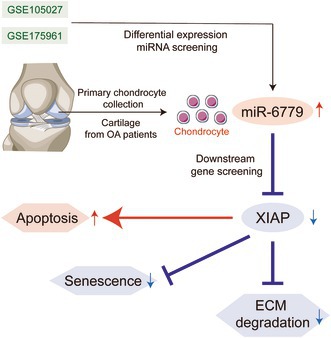

## 1 INTRODUCTION

Osteoarthritis (OA) is a degenerative joint disease that increases in prevalence with age, affecting the elderly population and impairing mobility due to joint pain. It is characterized by the aging of joint tissues, particularly cartilage, which leads to a decline in repair capacity and structural function.^1^, ^2^ As a result, joint replacement surgeries, such as knee arthroplasty, have become more common over the past 20 years.^3^ Chondrocytes, the unique cellular type in articular cartilage, are central to OA development and senescence. They maintain the extracellular matrix (ECM), which is crucial for the mechanics and structure of cartilage, which is primarily composed of type II collagen and aggrecan.^4^ Chondrocytes are also crucial for ECM synthesis and maintenance, and they obtain nutrients and signals from synovial fluid via synoviocytes in the synovial lining.^2^, ^5^ Senescence is defined by persistent cell cycle arrest, enhanced anti‐apoptosis ability, and secretion of damaging proinflammatory chemicals into the adjacent milieu; this is referred to as the senescence‐associated secretory phenotype (SASP).^6^ Accumulation of senescent cells with aging reduces cell proliferation and tissue homeostasis, function, and regeneration.^7^, ^8^ Senescence is linked to the pathogenesis of age‐related disorders like OA,^1^, ^5^ and researchers are exploring treatments for OA by targeting senescent chondrocytes and other joint cells.^9^, ^10^ Despite the correlation between aging, OA, and senescence, the exact mechanism connecting senescence to OA remains unclear.

Considering the specific features of SASP, another therapeutic method for treating OA is to target factors that induce damage and symptoms within this disorder, including pro‐inflammatory cytokines, chemokines, growth factors, matrix remodeling proteases (MMPs and ADAMTS), and the inhibitors of apoptosis proteins.^10^, ^11^, ^12^ At the epigenetic level, a microRNA (miRNA) with epigenetic‐regulatory features in gene expression modulation has been recently identified as a promising candidate for the treatment.^13^ MiRNAs are evolutionarily conserved, single‐stranded molecules of 20 to 22 nucleotides in length and function post‐transcriptionally by partial binding to the 3′‐untranslated region (UTR)s of mRNAs, resulting in mRNA destabilization and translation repression.^14^ In the case of OA, the activities of several critical signaling networks, including NF‐κB signaling,^15^, ^16^ Wnt/β‐catenin signaling,^17^ SIRT1/p53 signaling,^18^ p38MAPK signaling,^19^ and the SDF1/CXCR4 signaling,^20^ have been shown to be modulated by chondrocyte miRNAs implicated in OA development. Therefore, searching for differentially expressed miRNAs that could regulate chondrocyte senescence and apoptosis in OA might provide novel candidates for developing targeted therapies for OA.

In this study, differentially expressed miRNAs were analyzed based on the datasets GSE105027 and GSE175961, and the intersection was retrieved, disease target genes of OA were analyzed based on GeneCards, and potential miRNA‐target pairs were analyzed using RNAInter. Further, Metascape was used to identify miRNA‐target pairs related to cell proliferation/apoptosis/cycle, and miR‐6779 was obtained. Then, after isolating chondrocytes from OA joints, the expression of miR‐6779 and its specific functions in IL‐1‐induced chondrocytes were investigated. Based on Metascape analysis and RNAInter prediction, miR‐6779 binding and regulation of the X‐linked inhibitor of apoptosis protein (XIAP) were validated. Finally, the effects of XIAP alone and the dynamic effects of the miR‐6779/XIAP axis upon IL‐1‐induced chondrocytes were investigated. Collectively, the present study aims to demonstrate the role and mechanism of the miR‐6779/XIAP axis in reduced chondrocyte senescence and promoted apoptosis.

## 2 METHODS

### 2.1 Data source and tools

The datasets GSE105027 (containing 12 OA samples and 12 normal samples) and GSE175961 (containing 3 OA samples and 3 normal samples) were downloaded and analyzed for differentially expressed miRNAs in patients with OA and normal controls. OA disease target genes were retrieved from GeneCards (https://www.genecards.org/). Potential targets of miRNAs with high scores were predicted by RNAInter (http://www.rnainter.org/).^21^ Metascape (https://metascape.org/gp/index.html#/main/step1) was employed to perform functional and signaling pathway enrichment annotation.^22^

### 2.2 Chondrocyte isolation and identification

OA patients who underwent total knee replacement surgery between Jan 2022 and Sep 2022 were included. Samples of their cartilage were collected and the primary chondrocytes were isolated and cultured immediately. Briefly, the cartilage specimens were immediately placed in PBS to remove the soft tissues attached and rinsed twice with PBS. The thin layer of cartilage on the surface was cut off with a sterile surgical blade, and cartilage pieces about 15–20 mm^2^ in size were cut from the edge area of the severely degenerated cartilage area. The slices were again washed with 5 mL PBS and cut into pieces with ophthalmic scissors. The fragments were placed in a 15 mL centrifuge tube, and 0.15% type II collagenase was added at 10 times the volume of cartilage tissue. The centrifuge tube was placed in a constant temperature laboratory shaker at 37°C and 80 rpm and digested for 4–6 h. After isolating the chondrocytes, they were cultured with PBS or IL‐6 and Type II collagen was detected by immunofluorescent staining (IF) to identify the chondrocytes. Informed consent was obtained from all patients or guardians.

### 2.3 IF staining

Chondrocytes were cultured to 80% confluence before being transferred into serum‐free culture media for 24 h, followed by incubation in full culture media with PBS or IL‐1β (10 ng/mL). After being rinsed with PBS, cells were fixed in cold methanol for 20 min. Following a 10‐min permeabilization with 0.1% Triton X‐100, the cells were rinsed using PBS. The cells were then subjected to an overnight treatment with anti‐collagen II (Proteintech, Wuhan, China) at 4°C in PBS containing 1% BSA, followed by a 1‐h incubation at room temperature (RT) in the dark with a secondary Alexa Fluor® 488‐labeled antibody (green fluorescence, Cell Signaling Technology). After staining with DAPI (Thermo Fisher Scientific, blue fluorescence) for 5 min, cell nuclei were placed on glass slides and examined under a fluorescence microscope (Olympus, Japan).

### 2.4 qRT‐PCR

Total RNA was extracted from chondrocytes using TRIzol reagent. The concentration of RNA (Thermo Fisher Scientific, Waltham, MA, USA) was quantified using a NanoDrop ND‐2000 spectrophotometer. A PrimeScript RT reagent kit (Takara Bio) was utilized as per the instructions of manufacturers to reversely transcribe 1 μg of RNA. The real‐time quantitative PCR was conducted using an Applied Biosystems Real‐Time PCR system (Thermo Fisher Scientific). The 2^−ΔΔCt^ method was employed to examine the relative fold changes of target genes.^23^ GAPDH was set as an internal control. The primer sequence is listed in Table S1.

### 2.5 Cell transfection

Lipofectamine 3000 reagent (Invitrogen) was utilized following the instructions to transfect chondrocytes with 100 nM of NC antagomir, miR‐6779 antagomir, NC agomir, or miR‐6779 agomir. For XIAP knockdown or overexpression, short hairpin RNA targeting XIAP (sh‐XIAP‐1 or sh‐XIAP‐2, compared with sh‐NC) or XIAP‐overexpressing vector (compared with NC vector) was transfected into chondrocytes. IL‐1β or PBS was applied for 24 h to activate the transfected cells. The supernatants were collected.

### 2.6 Immunoblotting

Using the extraction reagent, proteins were extracted from NPCs. Proteins were dissolved in a loading buffer and measured using a BCA kit (Invitrogen). Following electrophoresis by 10% (w/v) sodium dodecyl sulfate‐polyacrylamide (SDS) gels, the separated proteins are electroblotted from the gel onto polyvinylidenefluoride (PVDF) membranes (Millipore, Billerica, MA, USA). Next, the proteins were incubated for 1 h at 37°C with 5% milk proteins in Tris‐buffered saline supplemented with 0.1% Triton X‐100. Then, the membrane was subjected to an overnight incubation at 4°C with primary antibodies against MMP3 (17837‐1‐AP, Proteintech, Wuhan, China), MMP13 (18165‐1‐AP, Proteintech), cleaved caspase 3 (9661, Cell Signaling Technology, MA, USA), cleaved‐caspase‐9 (9505, Cell Signaling Technology), XIAP (10037‐1‐Ig, Proteintech), P16 (bs‐23797R, Bioss, Beijing, China), P21 (AF6290, Affinity Bioscience, Changzhou, China), and GAPDH (AF7021, Affinity Bioscience). The secondary antibodies employed were goat polyclonal anti‐mouse IgG (H+L) horseradish peroxidase (HRP)‐labeled and goat anti‐rabbit IgG (H+L) HRP‐labeled (Proteintech). Electrochemiluminescence (ECL) reagent was used to visualize immunolabeling (Thermo Fisher Scientific).

### 2.7 Cell apoptosis

Cell apoptosis was detected using an annexin V‐FITC and PI apoptosis detection kit. Cells were transfected and then re‐suspended in 20 μL of binding buffer, followed by 5 μL of PI and 10 μL of annexin V‐FITC. Subsequently, samples were subjected to a 20‐min incubation in the dark. Flow cytometry (Novocyte, CA, USA) was applied to evaluate cell death.

### 2.8 Cell senescence

A staining kit was used to evaluate the activity of senescence‐associated β‐galactosidase (SA‐βGal) (Beyotime). Chondrocytes were plated onto 12‐well plates and fixed for 10 min at room temperature. Next, cells were rinsed, followed by overnight incubation at 37°C with the staining solution. ImageJ software (NIH) was used to quantify the SA‐βGal positive cell rates.

### 2.9 Dual‐luciferase reporter assays

Wild‐/mutant‐type XIAP reporter plasmids (wt‐/mut‐XIAP) were constructed based on a psiCheck‐2 plasmid (Promega); the putative miR‐6779 binding site in XIAP 3′UTR was mutated in mut‐XIAP. Wt‐/mut‐XIAP were co‐transfected to chondrocytes with the miR‐6779 agomir; luciferase activity was determined.

### 2.10 Statistical analysis

All data are expressed in terms of mean ± SD. SPSS 17.0 (IBM Corporation, Armonk, NY, USA) and GraphPad Prism 7.0 (GraphPad Software, CA, USA) were employed to analyze data. A *t* test was applied to contrast the differences between the two experimental groups. One‐way ANOVA was applied to analyze three groups or above. A *p* value of less than 0.05 was regarded as significant.

## 3 RESULTS

### 3.1 Differentially expressed miRNAs in OA cartilage and chondrocytes

**FIGURE 1:**
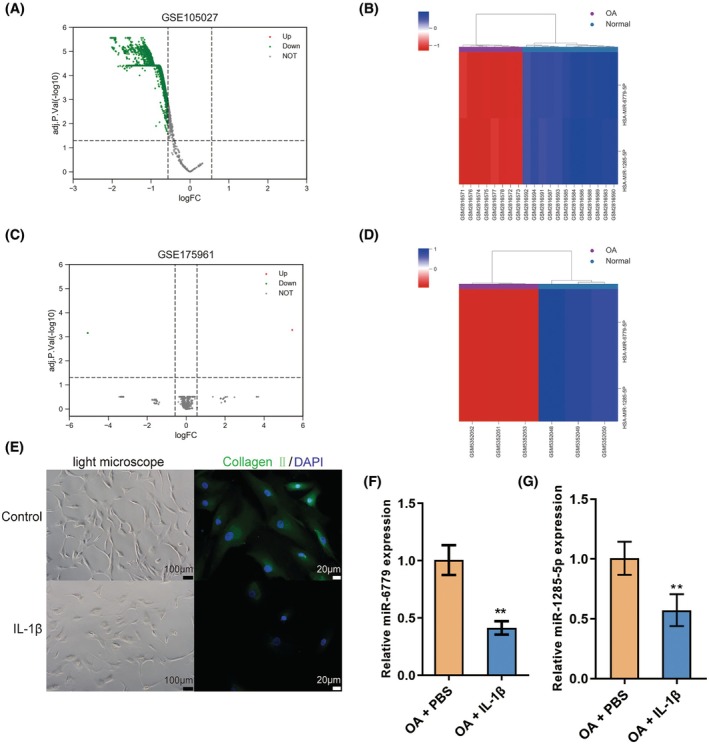
Differentially expressed miRNAs in osteoarthritis (OA) cartilage and chondrocytes (A, B) miRNAs differentially expressed in OA and normal samples based on GSE105027. (A) Volcano plot; (B) Hierarchical clustering heatmap. (C, D) miRNAs differentially expressed in OA and normal samples based on GSE175961. (C) Volcano plot; (D) Hierarchical clustering heatmap. (E) Chondrocytes were isolated from OA cartilage, treated with PBS or IL‐1β, and identified under an inverted microscope using immunofluorescent staining (IF) to detect collagen II (green fluorescence). The nucleus shows blue fluorescence. (F, G) Isolated chondrocytes were treated with PBS or IL‐1β and examined for the expression of miR‐6779 and miR‐1285‐5p using qRT‐PCR. **p < 0.01.

For identifying miRNAs with aberrant expression levels in OA cartilage, online datasets GSE105027 and GSE175961 were analyzed. By setting the thresholds of |logFC| >0.58, adj. *p* value <0.05, 2548 and 3 differentially expressed miRNAs were obtained from GSE105027 (Figure 1A,B) and GSE175961 (Figure 1C,D), respectively; by comparison, miR‐6779 and miR‐1285 were significantly down‐regulated in OA samples based on both datasets (Figure S1A–D). Next, chondrocytes were isolated from OA joints, stimulated with PBS or IL‐1β, and identified. IL‐1β induced decreases in the number of cells and the density of collagen II (Figure 1E). In response to IL‐1β treatment, qRT‐PCR was employed to determine miR‐6779 and miR‐1285‐5p expression levels. Both miRNAs were markedly down‐regulated within IL‐1β‐stimulated chondrocytes (Figure 1F,G).

Considering that miRNAs play their roles by binding to downstream mRNAs, miRNA targets and disease targets were analyzed and compared. Disease target genes of OA were analyzed based on GeneCards, and potential miRNA‐target pairs were analyzed using RNAInter; after comparison, 12 and 76 disease targets were found to be the targets of miR‐1285 and miR‐6779, respectively (Table 1). Furthermore, Metascape was used to identify miRNA‐target pairs related to cell proliferation/apoptosis/cycle. miR‐1285 targets were found to be enriched in cell migration, angiogenesis regulation, drug response, intracellular transport, cell cycle, external stimulation response regulation, and other biological processes (Figure S2A), while miR‐6779 targets were enriched in muscle cell proliferation, cell apoptosis signaling regulation, cell cycle, the FOXO pathway, the NF‐κB pathway, the P53 pathway, the PI3K‐Akt pathway, and the Ras pathway (Figure S2B,C), which were closely related to OA pathology. Therefore, miR‐6779 was chosen for the following investigations.

**TABLE 1:** The genes targeted by miR‐1285‐5p and miR‐6779‐5p.

miRNA	Target	miRNA	Target	miRNA	Target
hsa‐miR‐1285‐5p	SOD2	hsa‐miR‐6779‐5p	MATN3	hsa‐miR‐6779‐5p	MAZ
hsa‐miR‐1285‐5p	ADAMTS1	hsa‐miR‐6779‐5p	ADAMTS4	hsa‐miR‐6779‐5p	TMEM167A
hsa‐miR‐1285‐5p	PGF	hsa‐miR‐6779‐5p	TRAPPC2	hsa‐miR‐6779‐5p	CDKN1A
hsa‐miR‐1285‐5p	KLF2	hsa‐miR‐6779‐5p	COL5A1	hsa‐miR‐6779‐5p	SOX6
hsa‐miR‐1285‐5p	CEP250	hsa‐miR‐6779‐5p	ADIPOQ	hsa‐miR‐6779‐5p	CCL22
hsa‐miR‐1285‐5p	DDX55	hsa‐miR‐6779‐5p	IGF1	hsa‐miR‐6779‐5p	C3
hsa‐miR‐1285‐5p	PSMB8	hsa‐miR‐6779‐5p	MYH11	hsa‐miR‐6779‐5p	ATF6
hsa‐miR‐1285‐5p	RAN	hsa‐miR‐6779‐5p	FTO	hsa‐miR‐6779‐5p	AHR
hsa‐miR‐1285‐5p	LAMP1	hsa‐miR‐6779‐5p	NCOR2	hsa‐miR‐6779‐5p	YY1
hsa‐miR‐1285‐5p	PLS3	hsa‐miR‐6779‐5p	TRAPPC2B	hsa‐miR‐6779‐5p	CD3D
hsa‐miR‐1285‐5p	G3BP1	hsa‐miR‐6779‐5p	ITGB3	hsa‐miR‐6779‐5p	PFKFB3
hsa‐miR‐1285‐5p	GAS6	hsa‐miR‐6779‐5p	HAVCR2	hsa‐miR‐6779‐5p	FOXP4
hsa‐miR‐6779‐5p	DUSP19	hsa‐miR‐6779‐5p	TMEM91	hsa‐miR‐6779‐5p	MICB
hsa‐miR‐6779‐5p	XIAP	hsa‐miR‐6779‐5p	ZCCHC8	hsa‐miR‐6779‐5p	GNPTG
hsa‐miR‐6779‐5p	MBL2	hsa‐miR‐6779‐5p	HAUS3	hsa‐miR‐6779‐5p	SLC11A2
hsa‐miR‐6779‐5p	PDGFRA	hsa‐miR‐6779‐5p	TSKU	hsa‐miR‐6779‐5p	HEXA
hsa‐miR‐6779‐5p	MAT2A	hsa‐miR‐6779‐5p	EIF1AD	hsa‐miR‐6779‐5p	ARRB2
hsa‐miR‐6779‐5p	NFAT5	hsa‐miR‐6779‐5p	DNAH10OS	hsa‐miR‐6779‐5p	PLCE1
hsa‐miR‐6779‐5p	AGAP1	hsa‐miR‐6779‐5p	BMP3	hsa‐miR‐6779‐5p	CRTAP
hsa‐miR‐6779‐5p	KANSL1	hsa‐miR‐6779‐5p	IRAK3	hsa‐miR‐6779‐5p	CAPZB
hsa‐miR‐6779‐5p	TCTN2	hsa‐miR‐6779‐5p	FURIN	hsa‐miR‐6779‐5p	MBD6
hsa‐miR‐6779‐5p	RFT1	hsa‐miR‐6779‐5p	TNFRSF13C	hsa‐miR‐6779‐5p	BCL2L1
hsa‐miR‐6779‐5p	HNRNPUL1	hsa‐miR‐6779‐5p	GNAI3	hsa‐miR‐6779‐5p	F2RL2
hsa‐miR‐6779‐5p	RPRD2	hsa‐miR‐6779‐5p	DHODH	hsa‐miR‐6779‐5p	ALDOA
hsa‐miR‐6779‐5p	TYRO3	hsa‐miR‐6779‐5p	PGPEP1	hsa‐miR‐6779‐5p	YWHAZ
hsa‐miR‐6779‐5p	HMGA1	hsa‐miR‐6779‐5p	CSNK1D	hsa‐miR‐6779‐5p	GGCX
hsa‐miR‐6779‐5p	TRIB1	hsa‐miR‐6779‐5p	FOSL2	hsa‐miR‐6779‐5p	SMAD9
hsa‐miR‐6779‐5p	GCNT4	hsa‐miR‐6779‐5p	MDM2	hsa‐miR‐6779‐5p	CLEC7A
hsa‐miR‐6779‐5p	S1PR2	hsa‐miR‐6779‐5p	SLC16A4	hsa‐miR‐6779‐5p	GCFC2
hsa‐miR‐6779‐5p	TRAF3IP2				

### 3.2 Effects of miR‐6779 on IL‐1β‐induced chondrocyte dysfunction

**FIGURE 2:**
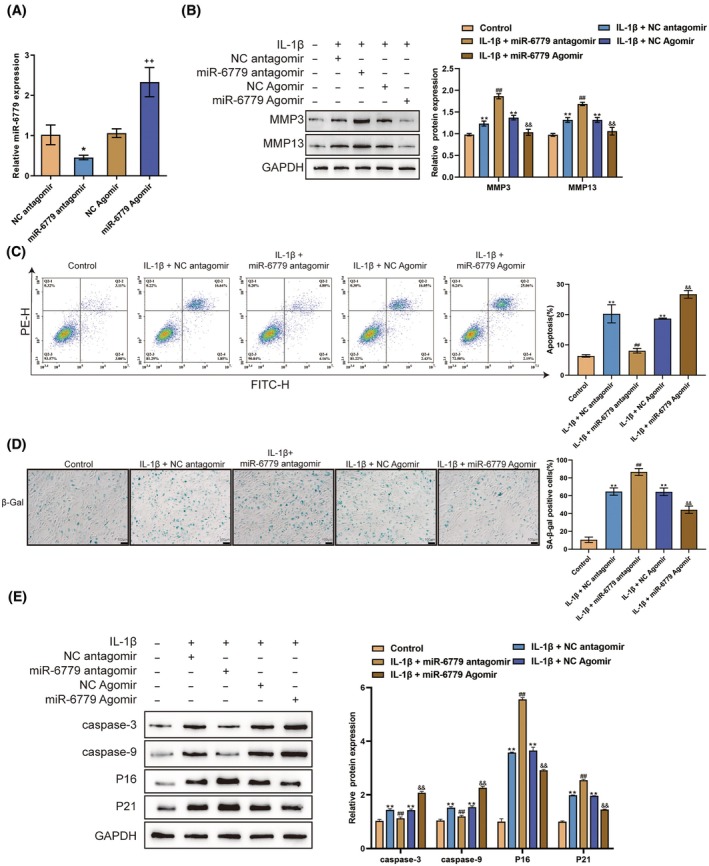
Effects of miR‐6779 on IL‐1β‐induced chondrocyte dysfunction (A) miR‐6779 expression was achieved by transfecting an miR‐6779 agomir/antagomir and validated by qRT‐PCR. Then, chondrocytes were transfected with an miR‐6779 agomir/antagomir, treated with PBS or IL‐1β, and examined for the protein levels of MMP3 and MMP13 using immunoblotting (B); cell apoptosis by flow cytometry (C); cell senescence by SA‐βGal staining (D); the protein levels of caspase‐3, caspase‐9, P16 and P21 using immunoblotting (E). **p < 0.01 versus control group; ^##^ *p* < 0.01 versus IL‐1β+ NC antagomir group; ^&&^ *p* < 0.01 versus IL‐1β+ NC Agomir group.

For investigating the specific effects of miR‐6779 upon OA chondrocytes, miR‐6779 overexpression or inhibition was achieved by transfecting an miR‐6779 agomir/antagomir and validated by qRT‐PCR (Figure 2A). Then, chondrocytes were transfected with an miR‐6779 agomir/antagomir, treated with PBS or IL‐1β, and assessed for cell phenotypes. IL‐1β stimulation considerably increased ECM degradation‐related MMP3 and MMP13 protein levels (Figure 2B), promoted cell apoptosis (Figure 2C) and cell senescence (Figure 2D), and elevated the levels of cleaved caspase‐3, caspase‐9 P16 and P21 (Figure 2E). miR‐6779 overexpression significantly reduced IL‐1β‐induced up‐regulation of MMP3 and MMP13 (Figure 2B), P16 and P21 expression and cell senescence (Figure 2D,E). Interestingly, miR‐6779 overexpression promoted cell apoptosis and expression of cleaved caspase‐3 and caspase‐9 (Figure 2C,E). miR‐6779 knockdown further enhanced IL‐1β‐induced MMP3 and MMP13 up‐regulation and senescence while reducing apoptosis. Therefore, miR‐6779 overexpression could increase OA chondrocyte apoptosis while reducing chondrocyte senescence and ECM degradation. These results indicated that miR‐6779 had different functions in chondrocyte apoptosis and senescence.

### 3.3 miR‐6779 targets and inhibits XIAP

**FIGURE 3:**
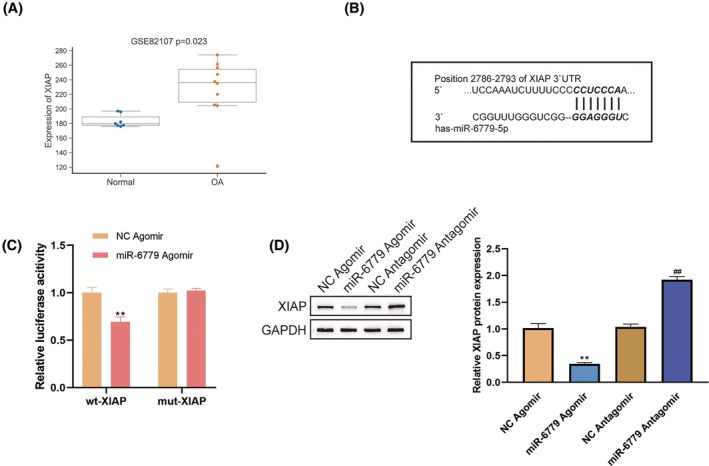
miR‐6779 targets and inhibits XIAP (A) XIAP expression in OA and normal samples according to GSE82107. (B, C) Dual‐luciferase reporter assays were performed. Wild−/mutant‐type XIAP reporter plasmids were constructed based on a psiCheck‐2 plasmid and co‐transfected to chondrocytes with an miR‐6779 agomir; luciferase activity was determined. (D) The expression of XIAP in response to miR‐6779 overexpression or knockdown was determined by immunoblotting. **p < 0.01 versus NC agomir; ^##^ *p* < 0.01 versus NC antagomir.

miR‐6779 targets are enriched in muscle cell proliferation, cell apoptosis signaling regulation, and cell cycle, which have been shown to be correlated with cell senescence; furthermore, miRNAs exert their functions through downstream targets. Therefore, miR‐6779 targets that are enriched in these processes were analyzed. Among 76 disease targets of miR‐6779, 5 genes were involved in apoptosis (Table S2), and XIAP and IGF1 were significantly up‐regulated in OA (Figures 3A and S1E). Moreover, under IL‐1β stimulation, XIAP mRNA levels showed a larger fold change (Figure 3B). Therefore, XIAP was chosen for further investigation.

For validating the predicted miR‐6779 binding to XIAP, dual‐luciferase reporter assays were performed. Wild−/mutant‐type XIAP reporter plasmids were constructed based on a psiCheck‐2 plasmid and co‐transfected to chondrocytes with an miR‐6779 agomir (Figure 3B); luciferase activity was determined. Figure 3C shows that miR‐6779 overexpression significantly suppressed the luciferase activity of wt‐XIAP but not that of mut‐XIAP. Moreover, in chondrocytes, miR‐6779 overexpression down‐regulated, whereas miR‐6779 inhibition up‐regulated XIAP expression (Figure 3D).

### 3.4 Effects of XIAP on IL‐1β‐induced chondrocyte dysfunction

**FIGURE 4:**
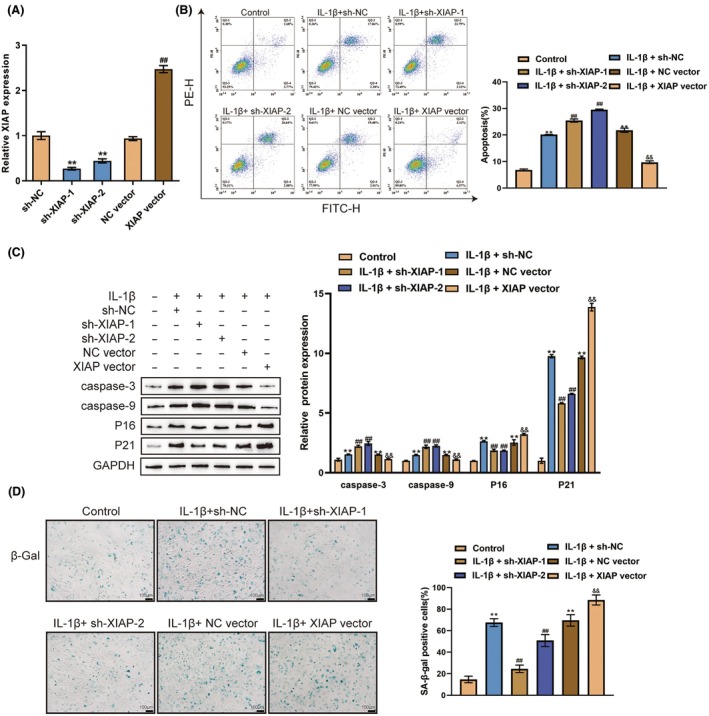
Effects of XIAP on IL‐1β‐induced chondrocyte dysfunction (A) XIAP expression was achieved by transfecting short hairpin RNA targeting XIAP (sh‐XIAP‐1/2) or XIAP‐overexpressing vector (XIAP vector) and validated by qRT‐PCR. Then, chondrocytes were transfected with sh‐XIAP‐1/2 or XIAP vector, treated with PBS or IL‐1β, and examined for: Cell apoptosis by flow cytometry (B); the protein levels of caspase‐3, caspase‐9, P16 and P21 using immunoblotting (C); cell senescence by SA‐β Gal staining (D). **p < 0.01 versus control group or sh‐NC group; ^##^ *p* < 0.01 versus IL‐1β + sh‐NC group or NC group; ^&&^ *p* < 0.01 versus IL‐1β+ NC vector group.

Concerning the specific effects of XIAP on chondrocytes, XIAP overexpression or knockdown was achieved by transfecting a short hairpin RNA targeting XIAP (sh‐XIAP‐1/2) or XIAP‐overexpressing vector (XIAP vector) and validated by qRT‐PCR (Figure 4A). Then, chondrocytes were transfected with sh‐XIAP‐1/2 or XIAP vector, treated with PBS or IL‐1β, and assessed for cell phenotypes. Similarly, IL‐1β stimulation dramatically promoted cell apoptosis (Figure 4B), elevated the levels of caspase‐3, caspase‐9, P16 and P21 (Figure 4C), and facilitated cell senescence (Figure 4D). XIAP knockdown significantly promoted cell apoptosis and reduced cell senescence, whereas XIAP overexpression showed opposite effects (Figure 4B–D). Therefore, XIAP knockdown could partially improve the senescence and dysfunction of chondrocytes induced by IL‐1β.

### 3.5 Dynamic effects of the miR‐6779/XIAP axis on chondrocytes

**FIGURE 5:**
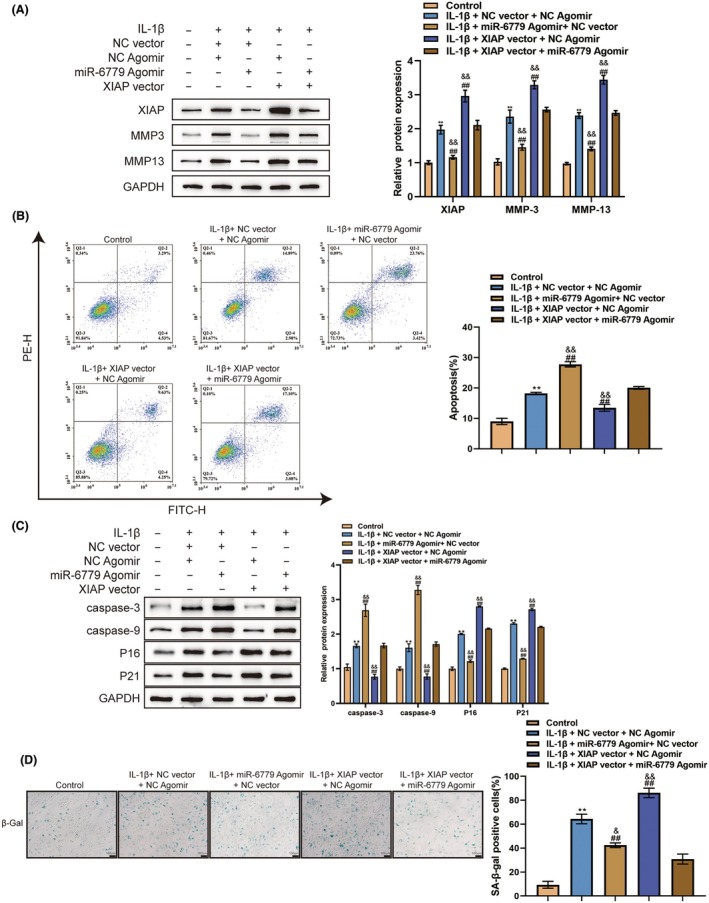
Dynamic effects of the miR‐6779/XIAP axis on chondrocytes (A) Chondrocytes were co‐transfected with an miR‐6779 agomir and XIAP vector, treated with PBS or IL‐1β, and examined for: The protein levels of MMP3 and MMP13 using immunoblotting (A); cell apoptosis by flow cytometry (B); the protein levels of caspase‐3, caspase‐9 P16 and P21 using immunoblotting (C); cell senescence by SA‐βGal staining (D). **p < 0.01 versus control group; ^##^ *p* < 0.01 versus IL‐1β+sh‐NC group; ^&&^ *p* < 0.01 versus IL‐1β+ XIAP vector +miR‐6779 agomir group.

The dynamic effects of the miR‐6779/XIAP axis upon chondrocytes were investigated to determine if miR‐6779 exerts its effects on chondrocytes through XIAP. Chondrocytes were co‐transfected with an miR‐6779 agomir and XIAP vector, treated with PBS or IL‐1β, and assessed for cell phenotypes. Under IL‐1β stimulation, miR‐6779 overexpression decreased the protein levels of MMP3 and MMP13 (Figure 5A), promoted cell apoptosis (Figure 5B), elevated caspase‐3 and caspase‐9 proteins (Figure 5C) and suppressed cell senescence and expression of P16 and P21 (Figure 5D); in contrast, under IL‐1β stimulation, XIAP overexpression increased MMP3 and MMP13 expression, cell senescence and senescent protein P16 and P21 levels while reducing cell apoptosis and expression of caspase‐3 and caspase‐9, which were partially reversed by miR‐6779 overexpression in chondrocytes (Figure 5A–D). These findings indicate that miR‐6779 targets and inhibits XIAP, thereby improving IL‐1β‐induced chondrocyte senescence and ECM degradation.

## 4 DISCUSSION

The present study used bioinformatics and experimental investigations to analyze and validate differentially expressed miRNAs in OA, which might affect chondrocyte apoptosis and senescence through target genes. miR‐6779 was found to be significantly down‐regulated in OA. The co‐targets of OA and miR‐6779 were enriched in cell proliferation, cell apoptosis, and cell cycle. miR‐6779 overexpression significantly attenuated IL‐1β effects on chondrocytes by reducing ECM degradation and cell senescence, while promoting cell apoptosis, and increasing caspase‐3 and caspase‐9 levels. miR‐6779 acts to inhibit XIAP expression. XIAP knockdown could partially improve IL‐1β‐induced chondrocyte senescence and ECM degradation. Lastly, when co‐transfected with an miR‐6779 agomir, the XIAP vector partially attenuated the effects of miR‐6779 overexpression on chondrocytes; miR‐6779 improved OA chondrocyte senescence and dysfunction through targeting XIAP.

miRNAs exert an essential effect on OA progression through various mechanisms, and chondrocyte senescence and dysfunction have been identified as key issues. Reportedly, the miR‐101/HRAS axis regulates IL‐1β‐triggered OA chondrocyte ECM breakdown, apoptosis, and senescence.^24^ miR‐27a serves as a regulator of the PI3K‐Akt–mTOR axis in human chondrocytes and could contribute to OA initiation through affecting chondrocyte autophagy and apoptosis.^25^ miR‐99b‐5p regulated MFG‐E8 to protect against OA by blocking senescence in chondrocytes and reprogramming macrophages through NF‐κB signaling.^26^ Herein, bioinformatics analyses based on datasets and experimental investigations indicated the down‐regulation of miR‐6779 and miR‐1285 in OA, suggesting their potential roles in OA pathogenesis. Furthermore, functional and signaling pathway enrichment annotation revealed that the co‐targets of OA and miR‐6779, but not miR‐1285, were enriched in muscle cell proliferation, cell apoptosis, cell cycle, and several inflammatory signaling pathways, which together define cell senescence.^6^ Therefore, miR‐6779 might modulate chondrocyte senescence to affect OA pathogenesis.

As previously mentioned, secretion of proinflammatory cytokines such as IL‐6, IL‐17, IL‐1, oncostatin M, and TNF^27^, ^28^ is a characteristic of the SASP, and multiple SASP components induce OA‐associated changes, including inflammation, bone growth, and ECM degradation. Pro‐inflammatory cytokines consequently increase the production of an enzyme family known as matrix metalloproteinases (MMPs), e.g. MMP13, and a disintegrin and metalloproteinase with thrombospondin motifs (ADAMTS), e.g. ADAMTS‐5.^29^ MMPs and ADAMTS released into the ECM could degrade ECM proteins in cartilage and cause cartilage ECM loss. ECM depletion, in turn, implicates chondrocyte senescence and other synovial joint cells as drivers of OA pathogenesis.^29^ Herein, IL‐1β treatment markedly increased MMP3 and MMP13 levels, whereas miR‐6779 overexpression partially decreased MMP3 and MMP13, suggesting that miR‐6779 overexpression partially attenuated IL‐1β‐induced ECM loss. Cytochemical β‐galactosidase activity staining remains a widely used method of measuring senescence within both cell culture and tissue specimens.^30^, ^31^ In this study, miR‐6779 overexpression also reduced the SA‐β‐gal‐positive chondrocyte number under IL‐1β stimulation. Correspondingly, the senescent biomarker P16 and P21^32^ levels were also reduced by miR‐6779 overexpression. Regarding cell apoptosis, it has been recognized that clearance of senescent cells by inducing apoptosis could attenuate the development of OA.^33^ Herein, miR‐6779 overexpression significantly facilitated chondrocyte apoptosis and elevated the levels of caspase‐3/9, suggesting that miR‐6779 could improve IL‐1β‐induced chondrocyte senescence and ECM loss via inducing apoptosis in senescent chondrocytes.

miRNAs exert their functions by targeting downstream mRNAs.^14^ Notably, in this study, XIAP and IGF1 were among the co‐targets of OA and miR‐6679. XIAP is a key member of the newly discovered family of IAP proteins and the strongest inhibitor of apoptosis in the IAP family.^34^ XIAP directly inhibits caspases and regulates apoptosis through various signaling pathways. IAP gene activation has been proven to provide apoptosis resistance to senescent cells and support senescent cell accumulation within tissues.^12^ In this study, miR‐6779 targeted and inhibited XIAP. Regarding its functions, XIAP knockdown facilitated chondrocyte apoptosis, inhibited chondrocyte senescence, and mitigated ECM loss under IL‐1β stimulation; more importantly, when co‐transfected with an miR‐6779 agomir, XIAP vector partially attenuated the effects of miR‐6779 overexpression upon IL‐1β‐stimulated chondrocytes, indicating that miR‐6779 exerts its actions on IL‐1β‐stimulated chondrocytes through targeting XIAP.

An increasing number of drugs have been found to target miRNAs that can alleviate OA recently. Qiu et al. found that exosomes derived from MSCs treated with curcumin could up‐regulate the expression of miR‐143 and miR‐124 in an OA in vitro model of chondrocytes, while inhibiting their apoptosis, thus delaying the progression of OA.^35^ Liu et al.'s study involved loading exogenous miR‐223 into extracellular vesicles derived from human umbilical cord mesenchymal stem cells and found that it inhibits activation of the NLRP3 inflammasome and chondrocyte pyroptosis, and produces great effects in the treatment of OA.^36^ Studies of miRNA in relation to OA have transcended the initial stages of merely examining phenomena and underlying mechanisms. Current research is increasingly focused on the efficacy of drug targets that interact with miRNA and the potential synergistic therapeutic impact when miRNA is integrated with exosomes and extracellular vesicles, marking these as trending areas of investigation.

## 5 CONCLUSION

miR‐6779 is down‐regulated, and XIAP is up‐regulated in OA cartilage and chondrocytes. miR‐6779 targets and inhibits XIAP, therefore promoting senescent chondrocyte apoptosis and reducing chondrocyte senescence and ECM loss through XIAP.

## AUTHOR CONTRIBUTIONS

**Zongchao Li:** Conceptualization; formal analysis; investigation; methodology; software; validation; visualization; writing – original draft; writing – review and editing. **Aonan Dai:** Investigation; writing – review and editing. **Xiaoxiang Fang:** Investigation; writing – review and editing. **Kexing Tang:** Investigation; writing – review and editing. **Kun Chen:** Data curation; writing – review and editing. **Peng Gao:** Data curation; writing – review and editing. **Jingyue Su:** Validation; writing – review and editing. **Xin Chen:** Validation; writing – review and editing. **Shengwu Yang:** Validation; writing – review and editing. **Zhenhan Deng:** Conceptualization; formal analysis; funding acquisition; methodology; project administration; resources; supervision; writing – review and editing. **Liangjun Li:** Conceptualization; formal analysis; funding acquisition; methodology; project administration; resources; supervision; writing – review and editing.

## FUNDING INFORMATION

The authors disclose receipt of the following financial or material support for the research, authorship, and/or publication of this article: National Natural Science Foundation of China (82472495), Natural Science Foundation of Hunan Province (2020JJ8043), Key Project of Hunan Provincial Health Commission (20201902 and C2019133), Natural Science Foundation of Changsha city, China (kq2403166), Hunan Provincial Clinical Medical Technology Innovation Guiding Project (2020SK53307), Basic Public Welfare Research projects of Wenzhou Science and Technology Bureau (Y20240087), and Start‐up Funding for Talented Scientific Research of the First Affiliated Hospital of Wenzhou Medical University (2023QD026).

## CONFLICT OF INTEREST STATEMENT

Zhenhan Deng is an editorial board member of Animal Models and Experimental Medicine (AMEM) and a corresponding author of this article. To minimize bias, he was excluded from all editorial decision making related to the acceptance of this article for publication.

## ETHICS STATEMENT

This study was approved by the Changsha Central Hospital Ethics Committee (No. R201911).

## Supporting information


Figure S1.

Figure S2.

Table S1.

Table S2.

## Data Availability

The data supporting this study's findings are available from the corresponding author upon reasonable request.
