# Supplementary material for: The miR‐6779/XIAP axis alleviates IL‐1β‐induced chondrocyte senescence and extracellular matrix loss in osteoarthritis
Source: Animal Model Exp Med. 2025 Feb 4;8(4):662–73. doi: 10.1002/ame2.12529 (PMC12008434; doi:10.1002/ame2.12529)
Supplement: Supplementary file 1 — Figure S1. [file AME2-8-662-s002.docx]

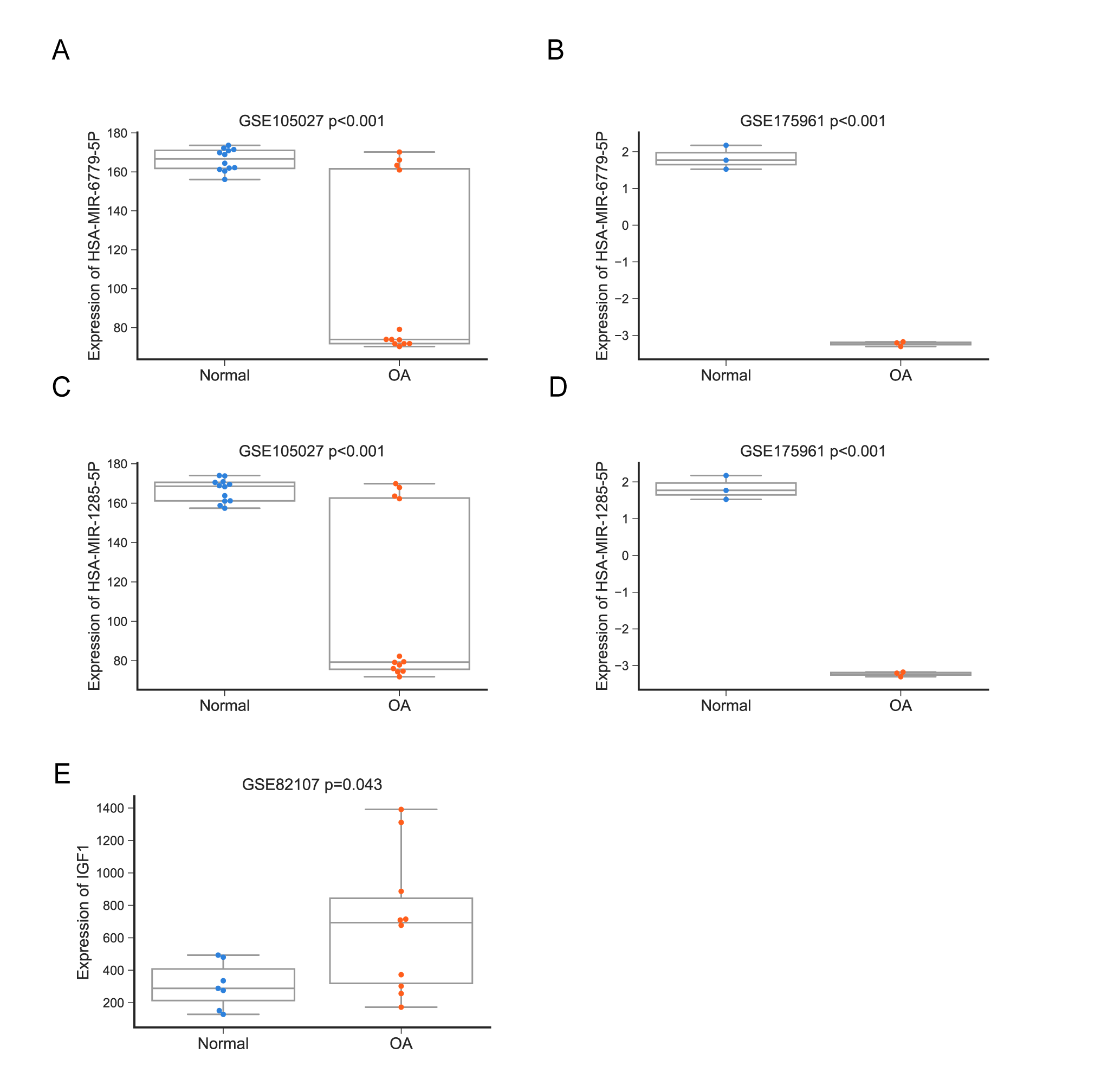


**Fig.S1 Expression of related factors in OA and normal samples according to online datasets** (A and C) The expression of miR-6779-5p and miR-1285-5p according to GSE105027. (B and D) The expression of miR-6779-5p and miR-1285-5p according to GSE175961. (E) The expression of IGF1 according to GSE82107.
