# Supplementary material for: The miR‐6779/XIAP axis alleviates IL‐1β‐induced chondrocyte senescence and extracellular matrix loss in osteoarthritis
Source: Animal Model Exp Med. 2025 Feb 4;8(4):662–73. doi: 10.1002/ame2.12529 (PMC12008434; doi:10.1002/ame2.12529)
Supplement: Supplementary file 2 — Figure S2. [file AME2-8-662-s001.docx]

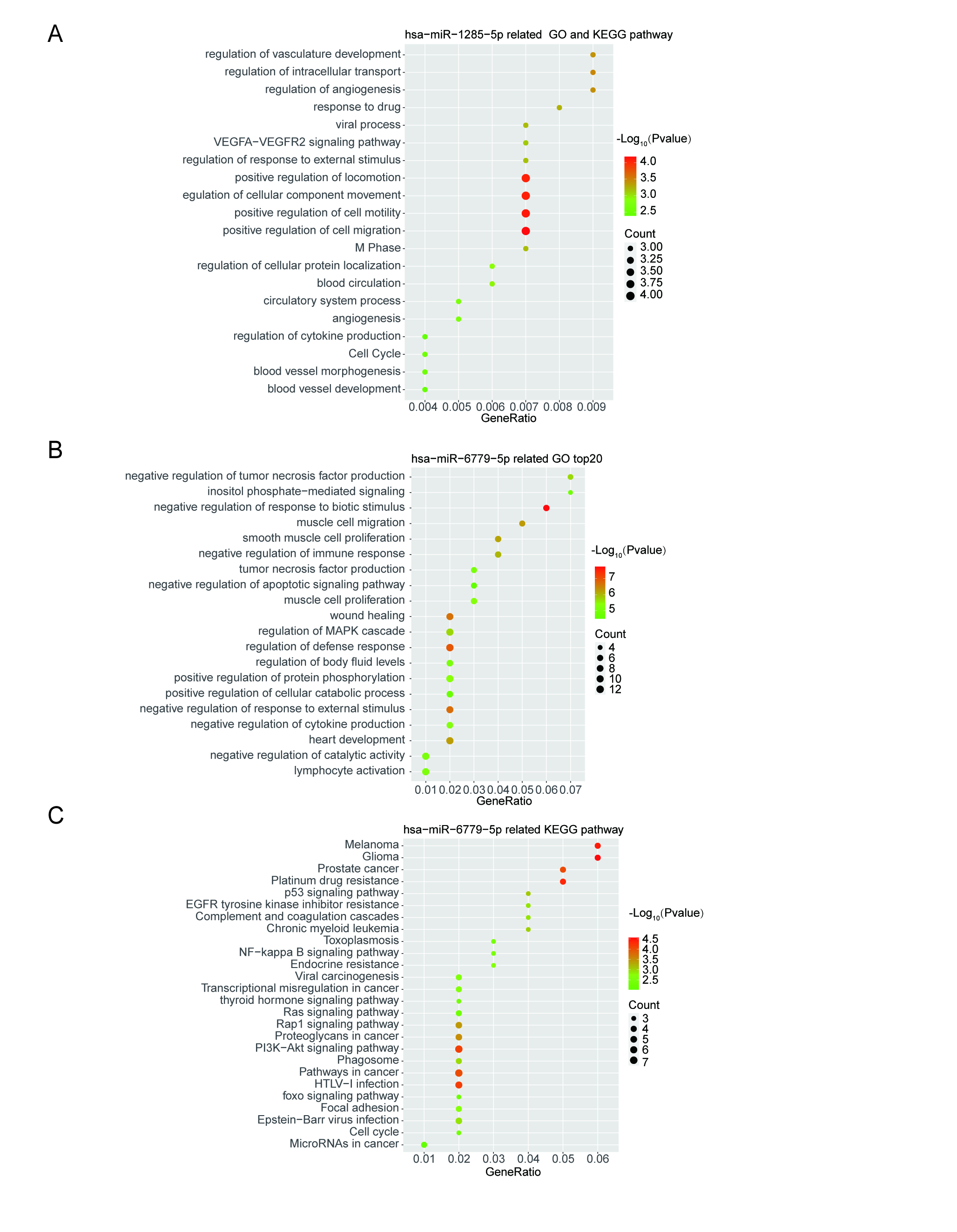


**Fig.S2 The GO and KEGG analysis of miR-1285-5p and miR-6779 targeted genes using Metascape.** (A) miR-1285-5p related GO and KEGG pathway. (B-C) miR-6779 related GO and KEGG pathway.
