# Supplementary material for: The miR‐6779/XIAP axis alleviates IL‐1β‐induced chondrocyte senescence and extracellular matrix loss in osteoarthritis
Source: Animal Model Exp Med. 2025 Feb 4;8(4):662–73. doi: 10.1002/ame2.12529 (PMC12008434; doi:10.1002/ame2.12529)
Supplement: Supplementary file 3 — Table S1. [file AME2-8-662-s003.docx]

Table S1 The primer sequence

| RT-qPCR | Forward | Reverse |
| --- | --- | --- |
| miR-6779 | RT：GTCGTATCCAGTGCGTGTCGTGGAGTCGGCAATTGCACTGGATACGACGCCAAAC  F:CTGGGAGGGGCTGG | CAGTGCGTGTCGTGGA |
| MiR-1285-5p | RT：GTCGTATCCAGTGCGTGTCGTGGAGTCGGCAATTGCACTGGATACGACCCTGGG  F:GCCGGATCTCACTTTGTTG | CAGTGCGTGTCGTGGA |
| U6 | CTCGCTTCGGCAGCACA | AACGCTTCACGAATTTGCGT |
| XIAP | AATAGTGCCACGCAGTCTACA | CAGATGGCCTGTCTAAGGCAA |
| GAPDH | ACAGCCTCAAGATCATCAGC | GGTCATGAGTCCTTCCACGAT |
| Sh-NC | GATCCGCAGATGAAGGCACGGTCACGCTCGAGGCAGATGAAGGCACGGTCACGTTTTTG | AATTCAAAAAGCAGATGAAGGCACGGTCACGCTCGAGGCAGATGAAGGCACGGTCACG |
| Sh-XIAP-1 | GATCCGGATATACTCAGTTAACAAGGCTCGAGCCTTGTTAACTGAGTATATCCTTTTTG | AATTCAAAAAGGATATACTCAGTTAACAAGGCTCGAGCCTTGTTAACTGAGTATATCCG |
| Sh-XIAP-2 | GATCCGCTACACAGTCATTACTTTCACTCGAGTGAAAGTAATGACTGTGTAGCTTTTTG | AATTCAAAAAGCTACACAGTCATTACTTTCACTCGAGTGAAAGTAATGACTGTGTAGCG |
| XIAP overexpression vector（plvx-puro） | ctaccggactcagatctcgagATGACTTTTAACAGTTTTGAAGGATCTAA | gtaccgtcgactgcagaattcTTAAGACATAAAAATTTTTTGCTTGAAA |
| NC Agomir | UUCUCCGAACGUGUCACGUTT | ACGUGACACGUUCGGAGAATT |
| MiR-6779 Agomir | CUGGGAGGGGCUGGGUUUGGC | CAAACCCAGCCCCUCCCAGUU |
| NC antagomir | CAGUACUUUUGUGUAGUACAA |  |
| MiR-6779 antagomir | GCCAAACCCAGCCCCUCCCAG |  |
| Wt-XIAP | aattctaggcgatcgctcgagATCACTCAGCACTCCAACTTCTAATC | attttattgcggccagcggccgcTACAAATAAAGATAAGTACTTATTGCAAAACTA |
| Mut-XIAP（mutant sits AAGAAAC） | CCCaagaaacAGAGTTCTCAGTGTCTACATGTAGACTATTCC | GAACTCTgtttcttGGGAAAAGATTTGGATATGGCTAT |
