# Supplementary material for: The miR‐6779/XIAP axis alleviates IL‐1β‐induced chondrocyte senescence and extracellular matrix loss in osteoarthritis
Source: Animal Model Exp Med. 2025 Feb 4;8(4):662–73. doi: 10.1002/ame2.12529 (PMC12008434; doi:10.1002/ame2.12529)
Supplement: Supplementary file 4 — Table S2. [file AME2-8-662-s004.docx]

**Table S2** 5 targeted genes of miR-6779 were involved in apoptosis pathway

| **Category** | **Description** | **Pvalue** | **InTerm_InList** | **Symbols** |
| --- | --- | --- | --- | --- |
| WikiPathways | Apoptosis | 8.80E-05 | 4/86 | XIAP,BCL2L1,IGF1,MDM2 |
| Reactome Gene Sets | Intrinsic Pathway for Apoptosis | 4.48E-04 | 3/55 | XIAP,BCL2L1,YWHAZ |
